# T‐Cell Lymphoma With Sarcoid‐Like Reaction and Secondary Myelofibrosis: A Unique Case Report and Literature Review

**DOI:** 10.1155/crh/6857208

**Published:** 2026-06-23

**Authors:** Renee Morecroft, Azalfa Lateef, Joseph Brandon Parker, Rachel Sauls, Jordan Phillipps, Patricia Chipi, Emily Wolf, Candido Rivera, Kirk Bourgeois, Benjamin Wang, Vikas Majithia, Sehreen Mumtaz

**Affiliations:** ^1^ Department of Internal Medicine, HCA Florida Orange Park Hospital, Orange Park, Florida, USA; ^2^ Department of Internal Medicine, Mayo Clinic, Jacksonville, Florida, USA, mayo.edu; ^3^ Department of Dermatology, Mayo Clinic, Jacksonville, Florida, USA, mayo.edu; ^4^ Department of Hematology and Medical Oncology, Mayo Clinic, Jacksonville, Florida, USA, mayo.edu; ^5^ Department of Laboratory Medicine and Pathology, Hematopathology, Mayo Clinic, Jacksonville, Florida, USA, mayo.edu; ^6^ Department of Rheumatology, Mayo Clinic, Jacksonville, Florida, USA, mayo.edu

**Keywords:** case report, sarcoid-like reaction, sarcoidosis, sarcoidosis-lymphoma syndrome, secondary myelofibrosis, T-cell lymphoma

## Abstract

**Introduction:**

T‐cell lymphomas are rare, comprising 10%–15% of non‐Hodgkin lymphomas (NHLs). Their etiology remains unclear, and pathophysiology varies widely among subtypes. Sarcoid‐like reactions (SLRs) have been reported in approximately 7.3% of NHLs, including T‐cell lymphomas, and can closely resemble sarcoidosis clinically/histologically. This overlap complicates diagnosis, particularly when secondary myelofibrosis—a rare and poorly understood complication—arises. Myelofibrosis has also been observed in sarcoidosis, further blurring the distinction between these entities. Treatment for T‐cell lymphoma‐associated secondary myelofibrosis is not standardized, with predominantly CHOP‐based regimens showing variable efficacy. This warrants further research to guide diagnostic and management frameworks.

**Case Description:**

We report the case of a 57‐year‐old male with known systemic sarcoidosis who developed recurrent symptomatic hypercalcemia and new‐onset transfusion‐dependent pancytopenia. Initial evaluation revealed bone marrow fibrosis with noncaseating granulomas, raising concern for sarcoidosis‐driven secondary myelofibrosis. However, repeat bone marrow biopsy demonstrated T‐cell lymphoma with a SLR. Primary myelofibrosis was excluded, supporting a secondary lymphoma‐driven process. The patient is currently undergoing treatment with BV‐CHP with close hematologic monitoring.

**Conclusion:**

T‐cell lymphoma‐associated SLR can closely mimic sarcoidosis and should be considered in the differential, particularly in patients with atypical presentations or cytopenias. Despite similar clinical and histological features, the underlying pathophysiology differs, necessitating thorough molecular and immunophenotypic evaluation. Though rare, this entity can lead to secondary myelofibrosis, underscoring the importance of excluding a primary myeloproliferative process. Further research is needed to define optimal therapy, with BV‐CHP representing a promising option in the evolving landscape of T‐cell lymphoma management.

## 1. Introduction

T‐cell lymphomas, a type of non‐Hodgkin lymphoma (NHL), are a rare and heterogeneous group of lymphoproliferative disorders arising from mature T‐cells. In 2025, the estimated incidence of NHL is 80,350 cases, with T‐cell lymphomas comprising 10%–15% of all NHLs and 20% of aggressive subtypes [1–3]. Although the etiology remains largely unclear, T‐cell lymphomas are believed to result from a complex interplay of genetic predisposition and environmental exposures, including viral infections such as Epstein–Barr virus (EBV) and human T‐cell leukemia Virus 1 (HTLV‐1) [1]. Genetic alterations in epigenetic regulators and key signaling pathway—such as JAK/STAT—are frequently implicated and contribute to aberrant T‐cell development, differentiation, and proliferation [4, 5]. The prognosis is generally poor, with most subtypes demonstrating a 5‐year overall survival rate around 30% [2]. Interestingly, T‐cell lymphoma can present with sarcoid‐like features—a phenomenon termed sarcoid‐like reaction (SLR)—which poses significant diagnostic challenges due to overlapping clinical and histopathological characteristics [6].

The co‐occurrence of sarcoidosis and lymphoma—particularly T‐cell lymphoma—has been previously described, referred to as sarcoidosis‐lymphoma syndrome (SLS) [7]. Sarcoid‐like granulomatous reactions have been observed in up to 13.8% and 7.3% of Hodgkin lymphoma and NHL patients, respectively [8]. SLS pathogenesis is thought to involve chronic immune activation and dysregulation [9]. In sarcoidosis, T‐helper cell and macrophage activation leads to granuloma formation, potentially fostering a microenvironment conducive to lymphomagenesis [9]. Prolonged immunosuppression, such as corticosteroid use, may further increase this risk [9]. The American Thoracic Society emphasizes the importance of excluding alternative diagnoses—including lymphoma—in patients with atypical clinical features or inadequate response to standard sarcoidosis therapy [10].

Clinically, sarcoidosis and T‐cell lymphoma may both present with lymphadenopathy, pulmonary infiltrates, fever, and weight loss, making differentiation difficult [8]. Histologically, sarcoidosis is defined by noncaseating granulomas, while SLR represents granulomatous inflammation that mimics sarcoidosis but does not meet its diagnostic criteria. In patients with known sarcoidosis, such findings may obscure an underlying lymphoma, necessitating thorough histopathological and immunohistochemical evaluation [8]. Notably, secondary myelofibrosis has been reported in both sarcoidosis and T‐cell lymphoma, though this overlap is rare and understudied. Bone marrow involvement in sarcoidosis is rare, with only a limited number of cases reported [11–14]. Similarly, SLR‐associated myelofibrosis in T‐cell lymphoma is exceedingly rare and underreported [15].

Clinically, sarcoidosis and T‐cell lymphoma may both present with lymphadenopathy, pulmonary infiltrates, fever, and weight loss, making differentiation difficult. The American Thoracic Society defines sarcoidosis diagnosis by three major elements: a compatible clinical/radiographic presentation, histologic evidence of non‐necrotizing granulomatous inflammation, and exclusion of alternative granulomatous diseases [16]. However, these criteria lack objective universal thresholds, making the diagnosis inherently uncertain and dependent on clinicopathologic correlation. This ambiguity is particularly relevant in patients with atypical features, cytopenias, marrow involvement, or poor response to sarcoidosis‐directed therapy, in whom lymphoma‐associated SLR should be excluded. Histologically, sarcoidosis is characterized by noncaseating granulomas, whereas SLR represents granulomatous inflammation that mimics sarcoidosis but occurs secondary to another process, including malignancy [16]. In patients with known sarcoidosis, these findings may obscure an underlying lymphoma, necessitating thorough histopathologic, immunophenotypic, and molecular evaluation.

CHOP‐based or CHOP‐like regimens, incorporating some combination of cyclophosphamide, doxorubicin, vincristine, and prednisone/prednisolone, are commonly used to treat T‐cell lymphomas; however, responses in T‐cell lymphoma‐associated secondary myelofibrosis have been variable.

Here, we describe a unique case of T‐cell lymphoma presenting as an SLR in a patient with systemic sarcoidosis, complicated by new‐onset transfusion‐dependent pancytopenia and bone marrow fibrosis. Initial findings, including bone marrow biopsy and negative flow cytometry, supported a diagnosis of sarcoidosis‐associated secondary myelofibrosis. However, further evaluation ultimately revealed underlying T‐cell lymphoma with secondary myelofibrosis. Uniquely, our case was treated with brentuximab vedotin in combination with cyclophosphamide, doxorubicin, and prednisone (BV‐CHP) in place of the typically reported CHOP regimen, with ongoing therapeutic monitoring. We contribute novel insight to the underreported field of T‐cell lymphoma‐associated SLR and secondary myelofibrosis, representing the first documented use of BV‐CHP in this context, while additionally providing a comprehensive literature review to further contextualize our case and highlight key diagnostic and management challenges associated with this rare clinical phenomenon.

## 2. Case Presentation

A 57‐year‐old male with systemic sarcoidosis initially diagnosed at a different institution (cutaneous, central nervous system, thyroid, and pulmonary involvement; on mycophenolate mofetil and prednisone) was admitted for severe, symptomatic hypercalcemia (corrected calcium 14.5 mg/dL; reference range, 8.5–10.5 mg/dL). He had experienced several similar but milder presentations in recent months, with hypercalcemia attributed to known sarcoidosis. During the current admission, initial management included intravenous fluids and calcitonin; however, clinical deterioration marked by worsening encephalopathy, diffuse bone pain, and tachycardia necessitated transfer to the ICU. There, he was treated with aggressive hydration, additional calcitonin, and zoledronic acid. His symptoms and calcium levels subsequently improved, allowing transfer back to the floor, where rheumatology recommended continuation of his immunosuppressive regimen.

His hospital course was complicated by new‐onset severe pancytopenia, prompting hematology consultation. The patient’s hemoglobin ranged from 6.1 to 8.7 g/dL (reference range, 13.5–17.5 g/dL), white blood cell count from 0.9 to 2.7 × 10^9^/L (reference range, 4.0–11.0 × 10^9^/L), and platelet count from 15 to 31 × 10^9^/L (reference range, 150–450 × 10^9^/L). Peripheral smear revealed a leucoerythroblastic picture without schistocytes. Coagulation studies, fibrinogen, D‐dimer, ADAMTS13 activity, and platelet Factor 4 antibody testing were all within normal limits. His anemia was hypoproliferative (Reticulocyte Index 1.1), with normal iron studies (ferritin 833 ng/mL [reference range, 30–400 ng/mL], iron saturation 77% [reference range, 20%–50%]), serum protein electrophoresis, and serum free light chain ratio. Workup for myeloproliferative neoplasms (JAK2, CALR, and MPL) and peripheral blood next‐generation sequencing was negative. CT imaging revealed mild splenomegaly (13 cm) with an interval decrease in mediastinal/hilar adenopathy and lung nodules compared to prior imaging. Bone marrow aspirate was inadequate because of the absence of marrow particles. Core biopsy demonstrated a hypercellular (“packed”) marrow with diffuse marked myelofibrosis (MF‐3 reticulin fibrosis), decreased trilineage hematopoiesis, noncaseating granulomata, and an atypical T‐cell infiltrate. Flow cytometry was negative for malignancy, and cytogenetics demonstrated a normal karyotype. Based on these findings—along with pancytopenia, leucoerythroblastosis, and splenomegaly in the absence of known MPN mutations—a provisional diagnosis of sarcoidosis‐associated secondary myelofibrosis was made.

**FIGURE 1:**
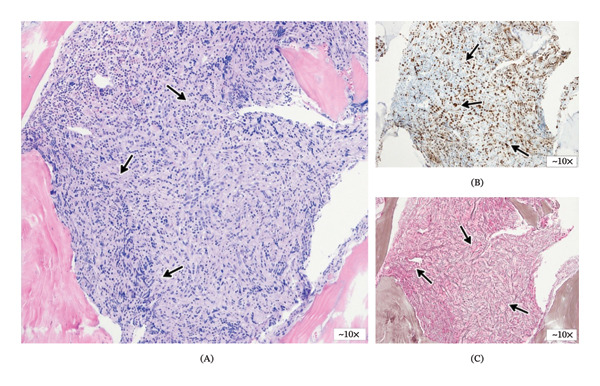
Bone marrow biopsy at 10x magnification demonstrating T‐cell lymphoma with sarcoid‐like reaction and secondary myelofibrosis. (A) Hematoxylin and eosin stain showing hypercellular fibrotic marrow with a vague swirling pattern. (B) Immunohistochemistry stain for CD3 showing relative increase in T‐cells, including a small subset of large, atypical T‐cells. (C) Reticulin stain showing diffuse fibrosis.

**FIGURE 2:**
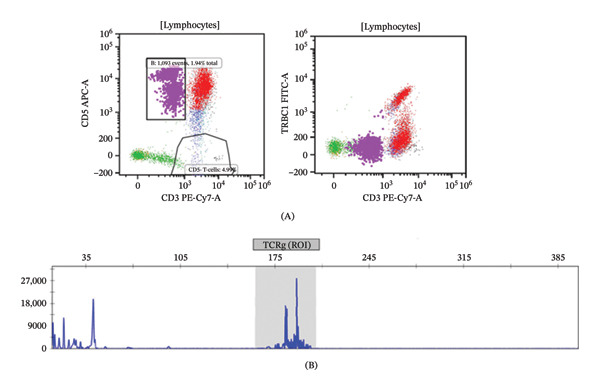
(A) Flow cytometry showing CD3 dim positive and TRBC1 negative. (B) T‐cell gene rearrangement (TCGR) showing probe set with prominent peaks.

The patient developed severe refractory thrombocytopenia requiring treatment with intravenous immunoglobulin and high‐dose corticosteroids. Mycophenolate mofetil was discontinued due to worsening pancytopenia, and adalimumab (40 mg every two weeks) was initiated but had a limited clinical response. Given the severity of his new transfusion‐dependent pancytopenia, leukoerythroblastosis, diffuse MF‐3 marrow fibrosis, limited response to sarcoidosis‐directed therapy, and atypical T‐cell infiltrate on the initial bone marrow biopsy, the case was rereviewed by hematopathology to exclude an occult lymphoproliferative process. Rereview of the bone marrow biopsy by hematopathology raised concern for T‐cell lymphoma with an SLR (Figure 1) rather than sarcoidosis‐associated secondary myelofibrosis. Repeat peripheral flow cytometry supported the diagnosis. Immunohistochemical studies showed atypical T‐cells positive for CD2, CD3, CD4, CD5, partial CD7, and CD45RO, with subset CD30 expression involving approximately 10% of CD3+ cells. The atypical cells were negative for CD8, CD123, CD303, and EBER. CD20 highlighted relatively decreased background B‐cells, while CD56 demonstrated rare scattered NK cells. CD34 and CD117 showed no increase in blasts. CD68 (PGM1) highlighted increased background marrow histiocytes, and MPO demonstrated decreased granulopoiesis. Special stains showed inadequate iron evaluation because of absent marrow particles, without evidence of ring sideroblasts. Flow cytometry of the bone marrow showed no increase in blasts and no monotypic B‐cell population. Peripheral blood flow cytometry identified an abnormal clonal CD4+ T‐cell population expressing CD2, dim/partial CD3, cytoplasmic CD3, CD4, CD5, partial CD7, CD10, and CD45, while lacking expression of CD8, CD16, TCRγ/δ, TRBC1, TdT, CD19, cCD22, cCD79a, and cMPO (Figure 2A). T‐cell gene rearrangement studies were positive for clonality (Figure 2B). Conventional cytogenetics demonstrated a normal karyotype. Myeloid next‐generation sequencing performed on the bone marrow was negative; however, peripheral blood sequencing identified a low‐level TET2 frameshift mutation (p.His955fs∗17) below the standard reportable variant allele frequency threshold (< 5%). Serum HTLV‐I/II testing was negative. Together, these findings established a monoclonal T‐cell process classified according to the 2022 WHO classification as peripheral T‐cell lymphoma (PTCL), not otherwise specified (PTCL‐NOS). The patient was started on combination systemic therapy with brentuximab vedotin (1.8 mg/kg), cyclophosphamide (750 mg/m^2^), doxorubicin (50 mg/m^2^), and prednisone (100 mg methylprednisolone equivalent for 5 days), administered every 21 days. His course remained complicated by persistent, transfusion‐dependent pancytopenia, requiring supportive care with filgrastim, romiplostim, and aminocaproic acid (for hematuria). Overall, he remains clinically stable with ongoing hospitalization for chemotherapy administration and close hematologic monitoring.

## 3. Discussion

We report a rare case of T‐cell lymphoma‐associated SLR and secondary myelofibrosis, initially presumed to be sarcoid‐driven myelofibrosis due to the patient’s known systemic sarcoidosis. T‐cell lymphomas may mimic sarcoidosis both clinically and histologically, particularly in cases of bone marrow fibrosis, posing significant diagnostic challenges. This diagnostic sequence has been previously described. Menghani et al. reported a patient initially diagnosed with sarcoidosis who was later found to have PTCL‐NOS, with retrospective biopsy review suggesting that the earlier presentation may have represented occult lymphoma rather than true sarcoidosis [17]. This similar case reinforces the importance of reconsidering lymphoma in patients with presumed sarcoidosis who develop atypical features, cytopenias, or poor response to sarcoidosis‐directed therapy. The literature on this overlap is scarce, and diagnostic/management strategies remain limited, complicating clinical decision‐making. Notably, we are the first to report BV‐CHP therapy for this rare clinical entity. Table 1 summarizes a focused literature review of T‐cell lymphoma‐associated secondary myelofibrosis, further contextualizing and distinguishing our findings.

**TABLE 1:** Summary of T‐cell lymphoma with secondary myelofibrosis cases and their response to chemotherapy.

Study title	Sample size	Study type	T‐cell lymphoma type	Chemotherapy	Response of fibrosis in bone marrow	Other key outcomes of study	Reference
^∗^Okabe et al. (2005)	1	Case report	PTCL with MF	THP‐OP	Progression on BMBX	Elevated TGF‐β1 may contribute to bone marrow fibrosis	[18]
^∗^Sekiguchi et al.2013	1	Case report	AITL with MF	CHOP	Complete response on BMBX	CNS recurrence without bone marrow recurrence	[19]
^∗^Jain et al. 2013	1	Case report	Primary autoimmune MF preceding high‐grade PTCL‐NOS	CHOP	Unknown	CNS progression on chemotherapy treated with intrathecal MTX but developed septic shock leading to death	[20]
^∗^Matsui et al. 2008	1	Case report	AITL with MF	CHOP	Complete response on FDG‐PET	Relapse with abdominal lymph node involvement treated with ESHAP and CHASE	[21]
^∗^Brenner et al. 1985	1	Case report	AILD with MF	n/a	n/a	Suggests a cytokine‐mediated process involving PDGF and TGF‐β	[22]
^∗^Saito et al. 1988	1	Case report	AITL with MF	CHOP	Complete response on BMBX	Early CNS recurrence without bone marrow involvement or fibrosis PDGF‐driven process	[23]
^∗^Kikukawa et al. 2009	1	Case report	PTCL with diffuse MF	n/a	n/a	The patient died from liver failure prior to receiving chemotherapy	[24]
^∗^Takai et al. 1994	1	Case report	PTCL with MF and CNS involvement	CHOP and intrathecal MTX	Complete response on BMBX after 3 months	n/a	[25]
^∗^Sekiguchi et al. 2015	1	Case report	PTCL‐NOS with MF	CHOP	Improvement of lymphoma and fibrosis after Cycle 1 but worsened after Cycle 2	n/a	[26]
^∗^Orth et al. 1994	1	Case report	AILD with MF	Vincristine and prednisone	Partial response on BMBX	n/a	[27]
^∗^Uehara et al. (2003)	1	Case report	PTCL with rapid progression of MF	Unknown regimen	Progression on BMBX	Improved symptoms but MF progressed, leading to death‐elevated TGF‐beta detected	[28]
^∗^Rao et al. (2003)	1	Case report	T‐cell lymphoma with MF	CHOP	Complete response on BMBX	Recurrence of lymphoma in the marrow after 4 cycles of CHOP treated with ICE	[29]
^∗^Abe et al. 2001	1	Case report	CTCL with MF	CHOP	Complete response on BMBX	High PDGF and TGF‐β levels detected	[30]
Weirich et al. (1998)	1	Case report	Hepatosplenic T‐gamma‐delta‐lymphoma with MF	CHOP	Unknown; CHOP discontinued due to postsplenectomy	Due to monoclonality DNA‐based molecular approach is a useful diagnostic tool	[31]
Sekiguchi et al. 2016	3	Case series	3 cases of PTCL	n/a	n/a	Positive for PDGF, TNF‐α, and/or TGF‐β suggesting a cytokine‐mediated process included a literature review of 13 other cases of T‐cell lymphoma with MF	[15]

*Note:* TGF‐β = tissue growth Factor 1; DNA = deoxyribonucleic acid; AITL = angioimmunoblastic T‐cell lymphoma; MF = myelofibrosis positive; TNF‐α = tumor necrosis alpha; MTX = methotrexate; CHOP = cyclophosphamide, doxorubicin, vincristine, and prednisolone; AILD = angioimmunoblastic lymphadenopathy; FDG‐PET = fluorodeoxyglucose‐positron emission tomography; ESHAP = etoposide, methyl‐prednisolone, cytosine arabinoside, and cisplatinum; CHASE = cyclophosphamide, etoposide, and dexamethasone; THP‐COP = pirarubicin hydrochloride, cyclophosphamide, vincristine, and prednisolone; BMBX = bone marrow biopsy.

Abbreviations: CNS = central nervous system; CTCL = cytotoxic T‐cell lymphoma; ICE = ifosfamide, carboplatinum, and etoposide; NOS = not otherwise specified; PCR = polymerase chain reaction; PDGF = platelet‐derived growth factor; PTCL = peripheral T‐cell lymphoma.

^∗^Cases included in Sekiguchi et al.’s study, 2016.

SLS, first described in 1986, refers to the coexistence of sarcoidosis and lymphoproliferative disorders [7]. Lymphoma may develop years after a sarcoidosis diagnosis—sometimes as early as 1 year or as late as several decades—and can also precede or coincide with sarcoidosis [9, 16]. Middle‐aged individuals with chronic, active sarcoidosis are at increased risk for lymphomas, particularly low‐grade subtypes with pulmonary origin [9]. While other hematologic malignancies, such as leukemia and multiple myeloma, have been reported in patients with long‐standing sarcoidosis, lymphoma remains the most frequent and clinically relevant association [9, 17].

Although sarcoidosis and T‐cell lymphoma‐associated SLR share immunologic mechanisms—especially the formation of noncaseating granulomas—they differ in etiology and downstream marrow effects. Sarcoidosis represents an exaggerated immune response to unidentified environmental or infectious antigens in genetically predisposed individuals. It is characterized by well‐formed, noncaseating granulomas composed of CD4+ T‐helper cells and macrophages, driven by a Th1‐polarized cytokine profile (e.g., IL‐2, IFN‐γ, TNF‐α, and TGF‐β) [32]. While this inflammatory milieu may contribute to fibrosis, clinically significant bone marrow fibrosis is uncommon [32].

In contrast, SLR in T‐cell lymphoma reflects an immune response to tumor‐associated antigens, with sustained antigenemia, macrophage activation, and cytokine‐mediated histiocyte recruitment [9]. Importantly, malignant T‐cells may secrete profibrotic cytokines—including IL‐6, IL‐10, CXCL13, VEGF, and TGF‐β—which can directly stimulate fibroblast proliferation and extracellular matrix deposition within the bone marrow [9]. This provides a mechanistic link between lymphoma‐associated immune dysregulation and the development of secondary myelofibrosis.

Prior reports, including that of Da Silva Constante et al., describe PTCL associated with cytokine‐driven secondary myelofibrosis in the setting of clonal T‐cell proliferation [33]. Similarly, in our case, the progression from granulomatous inflammation to diffuse marrow fibrosis, coupled with T‐cell clonality, supports a lymphoma‐driven rather than sarcoidosis‐associated process.

Distinguishing sarcoidosis from lymphoma‐associated SLR is difficult due to overlapping features such as noncaseating granulomas. However, histologic differences can aid diagnosis. In sarcoidosis, granulomas are typically well‐formed and non‐necrotizing, composed of epithelioid histiocytes and multinucleated giant cells, and rimmed by CD4+ T‐cells and occasional B‐cells [10, 32]. In contrast, granulomas in lymphoma‐associated SLR may be located within or near tumor sites and are often accompanied by atypical lymphoid cells [8, 34].

The presence of a monoclonal T‐cell population—detectable via T‐cell receptor gene—is a key differentiator, as sarcoidosis typically shows polyclonal T‐cells [31, 34]. The British Society for Hematology outlines three major criteria for myelofibrosis: (1) bone marrow fibrosis grade ≥ 3, (2) a driver mutation (e.g., *JAK2, CALR,* or *MPL*), and (3) palpable splenomegaly—along with at least one minor criterion, such as unexplained anemia, leukoerythroblastic blood film, dacrocytes, constitutional symptoms, or extramedullary hematopoiesis [35]. Differentiating primary from secondary myelofibrosis is critical for guiding therapy. The National Comprehensive Cancer Network (NCCN) also highlights the prognostic value of bone marrow histopathology and features such as circulating CD34+ cells, clonal hematopoiesis, and JAK2 mutations in diagnosing primary myelofibrosis [36]. In our case, these features were absent, supporting a diagnosis of secondary myelofibrosis.

Bone marrow involvement in both sarcoidosis and T‐cell lymphoma is rare but can lead to fibrosis and cytopenias [10, 13]. Four prior reports described bone marrow sarcoidosis with noncaseating granulomas and fibrosis [11–14]. 18F‐FDG PET imaging can help detect marrow involvement, and corticosteroids have been shown to reduce granuloma burden and improve hematologic indices [13]. The mechanisms underlying T‐cell lymphoma‐associated myelofibrosis are less well understood but are thought to be cytokine‐mediated, akin to primary myelofibrosis [15]. Key mediators include platelet‐derived growth factor, TGF‐β, and TNF‐α (15,24,25). Proposed mechanisms include the following: [1] direct cytokine secretion by infiltrating lymphoma cells, [2] paracrine signaling from distant sites, or [3] a combination of both [15]. To date, 17 cases of T‐cell lymphoma‐associated myelofibrosis have been reported, including angioimmunoblastic T‐cell lymphoma (*n* = 8), PTCL (*n* = 6), cytotoxic T‐cell lymphoma (*n* = 1), unspecified T‐cell lymphoma (*n* = 1), and hepatosplenic T‐gamma‐delta‐lymphoma (*n* = 1) (Table 1) [15, 31]. Our case adds to this growing body of literature.

No standardized treatment for T‐cell lymphoma‐associated myelofibrosis exists. However, addressing the underlying lymphoma often leads to fibrosis resolution [29]. Of the above cases, nine were treated with CHOP; one received pirarubicin hydrochloride, cyclophosphamide, vincristine (THP‐OP), and prednisolone; and another received vincristine and prednisolone alone (Table 1) [18–21, 23, 25–27, 29, 31]. THP‐OP and vincristine/prednisolone led to disease progression or only partial response [18, 27]. Among those treated with CHOP, six achieved complete fibrosis resolution; one showed initial improvement followed by progression [19, 21, 23, 25, 26, 29, 30]. The ECHELON‐2 trial, a global, double‐blind, randomized Phase 3 study, compared BV‐CHP to standard CHOP in previously untreated CD30‐positive PTCL [37]. BV‐CHP demonstrated significantly longer median progression‐free survival (48.2 vs 20.8 months; HR 0.71, 95% CI 0.54–0.93; *p* = 0.0110) and improved overall survival (HR 0.72, 95% CI 0.53–0.99) [37]. Safety profiles were similar between regimens, with comparable rates of febrile neutropenia and peripheral neuropathy. These results support BV‐CHP as a superior first‐line treatment for CD30‐positive PTCL. Given these findings, BV‐CHP may represent a promising treatment for T‐cell lymphoma with secondary myelofibrosis. Ours is the first reported case using BV‐CHP in this setting, with promising initial response and ongoing monitoring.

Here, we present a unique case of T‐cell lymphoma‐associated SLR and secondary myelofibrosis in a patient with known sarcoidosis, treated with BV‐CHP. Our case highlights the diagnostic complexity and therapeutic challenges posed by overlapping clinical and pathologic features, underscoring the importance of maintaining a high index of suspicion, incorporating molecular testing, and engaging multidisciplinary teams. Furthermore, it emphasizes the need to evaluate for secondary myelofibrosis in patients with new‐onset cytopenias, particularly when myeloproliferative mutations are absent. Given the rarity of this clinical entity, our reports offer a comprehensive diagnostic and management framework contextualized within the current literature to enhance clinical recognition and guide diagnostic workup. Larger, prospective studies are warranted to improve risk stratification, refine diagnostic criteria, and establish standardized management approaches for this rare but challenging condition.

**Nomenclature**
TNFα: Tumor necrosis factor‐alpha
SLR: Sarcoid‐like reaction
SLS: Sarcoidosis‐lymphoma syndrome
EBV: Ebstein–Barr virus
MPN: Myeloproliferative neoplasm
JAK2: Janus Kinase‐2
CALR: Calreticulin
MPL: Myeloproliferative leukemia virus
BV‐CHP: Brentuximab vedotin combined with cyclophosphamide, doxorubicin, and prednisone
CHOP: Cyclophosphamide, doxorubicin, vincristine, and prednisolone
IL: Interleukin
TGF‐β: Transforming growth factor‐beta
PTCL: Peripheral T‐cell lymphomas

## Author Contributions

Conceptualization: Vikas Majithia and Sehreen Mumtaz. Investigation: Azalfa Lateef, Joseph Brandon Parker, Rachel Sauls, Jordan Phillipps, Patricia Chipi, Emily Wolf, Candido Rivera, Kirk Bourgeois, and Benjamin Wang. Visualization: Renee Morecroft and Jordan Phillipps. Writing–original draft: Renee Morecroft and Jordan Phillipps. Writing–review and editing: all authors. Supervision: Vikas Majithia and Sehreen Mumtaz. Dr. Sehreen Mumtaz served as manuscript guarantor. Dr. Vikas Majithia had full access to all of the data in this study and takes complete responsibility for the integrity of the data and the accuracy of the data analysis.

## Funding

This article has no funding source.

## Disclosure

All authors have read and approved the final version of the manuscript.

## Ethics Statement

This case report was conducted in accordance with the principles outlined in the Declaration of Helsinki. All identifying information has been removed to protect patient confidentiality. Informed consent was obtained from the patient for publication of this case and any accompanying images. Institutional Review Board approval was not required.

## Consent

A written informed consent was obtained from the patient for publication of this case report and any accompanying images.

## Conflicts of Interest

The authors declare no conflicts of interest.

## Data Availability

The data that support the findings of this study are available from the corresponding author upon reasonable request.
